# Synergetic-PI controller based on genetic algorithm for DPC-PWM strategy of a multi-rotor wind power system

**DOI:** 10.1038/s41598-023-40870-7

**Published:** 2023-08-21

**Authors:** Habib Benbouhenni, Hamza Gasmi, Ilhami Colak, Nicu Bizon, Phatiphat Thounthong

**Affiliations:** 1 Department of Electrical and Electronics Engineering, Faculty of Engineering and Architecture, Nisantasi University, 34481742 Istanbul, Turkey; 2 Laboratoire Controle Avancé (LABCAV), Department of Electronics and Telecommunications, University of Guelma, BP 401, 24000 Guelma, Algeria; 3 Faculty of Electronics, Communication and Computers, University of Pitesti, 110040 Pitesti, Romania; 4 ICSI Energy, National Research and Development Institute for Cryogenic and Isotopic Technologies, 240050 Râmnicu Vâlcea, Romania; 5 Doctoral School, Polytechnic University of Bucharest, 313 Splaiul Independentei, 060042 Bucharest, Romania; 6 Faculty of Technical Education, Renewable Energy Research Centre (RERC), King Mongkut’s University of Technology North Bangkok, 1518 Pracharat 1 Road, Wongsawang, Bangsue, Bangkok, 10800 Thailand

**Keywords:** Energy science and technology, Engineering

## Abstract

This work designs a powerful new nonlinear control technique using synergetic control (SC), proportional-integral (PI) controller, and genetic algorithm (GA) for multi-rotor wind energy (MRWE) conversion systems, whereby an asynchronous generator (AG) is used to achieve optimal energy extraction. The direct power control (DPC) technique is used based on the proposed SC-PI-GA (SPI-GA) technique to control the AG-based MRWE system, where this new nonlinear control technique is used to achieve stable control characteristics under random changes in wind speed and to provide great robustness against modeling uncertainties. Moreover, the pulse width modulation (PWM) technique is used to control the AG inverter due to its simplicity and ease of implementation. In this proposed DPC-SPI-GA technique, we need to measure current and voltage to estimate the active power and the reactive power. Also, inner loops are not used in this proposed DPC-SPI-GA technique as is the case in the field-oriented control (FOC) technique, where the proposed system in this work is characterized by an integrated structure. Three different tests are proposed to study and verify the behavior of the designed DPC-SPI-GA strategy compared to the traditional DPC technique.

## Introduction

Traditionally, the asynchronous generator (AG) is a type of electric generator that has become famous in recent years in the field of renewable energies, especially in wind energy (WE) due to its durability, ease of control, low cost, and low maintenance compared to other types^1,2^. Also, this type of generator has an attractive feature that distinguishes it from the rest, as it has less power loss and the variable speed operation with the excitation transformer is only 25–35% of the generator rating^3^. The wind system that uses the AG is characterized by low cost and can be operated in the case of variable wind speed^4^. But among its downsides is that it is very sensitive to network disturbances due to the direct contact between the generator stator and the network, and the rotor-side excitation transformer has a limited power rating^5^. As is well known, depending on the wind speed, the power generated by the generators changes. Induction generators are electrical machines that can operate as generators or as motors. Therefore, these electric generators can operate as motors and become consuming electrical energy in this case, and this is not desirable. Accordingly, if the wind speed is less than the limit in which the machine operates as an electric generator, the turbines are stopped.

In the field of WE, several types of turbines can be used to generate enough energy from the wind to spin a generator. These types can be divided into two types: single-rotor WEs^6–8^ and multi-rotor WEs^9,10^. Whereas the multi-rotor WE are a development of the single-rotor WE to increase the value of torque and mechanical energy required to rotate the generator^11^. In addition, the use of multi-rotor WEs leads to a reduction in the area of wind farms and overcomes the winds generated by turbines in wind farms^12^.

In multi-rotor WEs, it is possible to use two turbines rotating on the same axis and being at a distance, and we can find two turbines at the same point and rotating in the opposite direction, depending on the technology used. In recent years, a new technology of multi-rotor turbines containing 4 or 6 turbines has emerged to greatly increase the energy gained from the wind and to exploit the technology to a large extent to gain space for wind farms and reduce the cost of energy production and the use of traditional sources of energy. For electric power generation. In addition, the energy gain from wind in the case of multi-rotor WEs is greater than the energy gain from wind in the case of conventional single-rotor turbines. Also, the system based on multi-rotor WEs is more stable and is not affected by the wind generated between the turbines in the wind farms^10^. The negative of this technology is that it contains a large number of mechanical components, which makes it expensive and costly during periodic maintenance^9^. These technology (multi-rotor WEs) are complex and difficult to control compared to conventional single-rotor turbines.

In the field of controlling electric generators, three control techniques are more prevalent in the field of generating electric power from wind, where they are widely used in controlling AGs because of the simplicity and ease of control. These control techniques are represented in field-oriented control (FOC)^13^, direct torque control (DTC)^14,15^, and direct power control (DPC)^16^, where there are differences and similarities between these three control techniques. DPC and DTC techniques are among the most famous and most widely used controls in the field of controlling electrical machines due to their simplicity, ease, and fast dynamic response. These controls are linear controls, where these strategies depend on estimating the characteristic values using measuring voltage and current. In the FOC strategy, inner loops are used and depend on the use of the proportional-integral (PI) controller to control the active and reactive power of the AG-based wind turbine system^17^. This strategy relies on the use of pulse width modulation (PWM) to generate the inverter control pulses. There are two types of this control, the direct FOC strategy^18^ and the indirect FOC strategy^19^, and the difference between them lies in the number of PI controllers used. FOC is characterized by several drawbacks that make it among the unsatisfactory or reliable solutions, as high power ripples and low current quality are among the most prominent drawbacks of this strategy. Also, the slow dynamic response is a drawback of this strategy that limits its spread in the control field. On the other hand, the DTC technique is very different from the FOC technique in terms of principle and degree of complexity^20^. The DTC technique is characterized by durability and simplicity compared to the FOC strategy, where a switching table is used to control the generator inverter and two hysteresis controllers are used to control the reactive and active power (*Qs* and *Ps*)^21^. This technique was used to control several electrical machines^22–25^. In addition, the DPC technique is very similar to the DTC technique in its working principle, and the difference between them lies in the references used^26^. In the DPC, both the *Qs* and *Ps* are used as references, and the flux and torque are used as references in the DTC technique. Because of the simplicity of the algorithm, ease of implementation, and the lack of inner loops, The DPC technique has been proposed as the best solution for controlling the AG-based wind turbine^27^. Moreover, this technique has a faster dynamic response compared to the FOC strategy^28^. The DPC and DTC strategies depend on estimation to a large extent, as the estimation process is indispensable for distinct quantities. To conduct the assessment in the two controls, both voltage and current must be measured, as voltage and current measuring devices of high efficiency must be used to reduce the error in the distinguished amounts and thus obtain good results. In this paper, we will focus on the DPC technique due to its features and the importance of the issue of electric power generation from wind energy, where a new idea for the DPC technique and a new system for electric power generation based on the use of a multi-rotor wind turbine (MRWT) is proposed.

As is well known, any control technique has advantages and disadvantages that distinguish it from other techniques. The DPC technique has many drawbacks, including the presence of fluctuations at the level of torque, reactive and active power, high total harmonic distortion (THD) value of current, and low quality of energy^29^. Several researchers tried to improve the characteristic of the DPC technique for the AG-WE system and overcome the negatives by using several techniques such as artificial intelligence techniques. In Ref.^30^, the author used sliding mode control (SMC) to improve the characteristic of the DPC technique used to control an AG-based wind turbine, where experimental results proved the effectiveness of the DPC-SMC strategy in tracking references compared to the DPC. But the negative of using the SMC strategy is that it is related to the studied system, which makes its application more complicated. Moreover, if the system parameters are changed, the DPC-SMC strategy is affected, which causes the current quality to drop significantly, which is not desirable. In Ref.^31^, the rotor current feedback controller is used to increase the efficiency of the DPC strategy for an AG-based wind turbine system. Compared with the experimental results of the classical DPC technique, the DPC technique based on a rotor current feedback controller is characterized by dynamic speed and lower active power ripples. In Ref.^32^, the second-order SMC (SOSMC) technique and DPC strategy are combined to control the asynchronous generator. The DPC-SOSMC technique is characterized by durability, as it is noted that the switching table is not used to control the generator inverter. Also, note that hysteresis comparators are not used in the proposed DPC-SOSMC technique. The DPC-SOSMC technique shows superior characteristics of control in terms of power ripple elimination compared to the DPC technique. In Ref.^33^, the author introduces a new idea for the DPC technique using a voltage-oriented stator control to minimize the torque and current ripples of the AG-WE system. The designed control scheme is different from the classical method, where satisfactory results were obtained in terms of active power fluctuations and current quality. In Ref.^34^, an adaptive-gain SOSMC-direct power command (AGSOSMC-DPC) is used to control an AG, valid for both balanced and unbalanced grid voltage. This proposed AGSOSMC-DPC strategy is characterized by stability, as the stability of the proposed AGSOSMC-DPC technique was confirmed using Lyapunov's theory. Also, the proposed AGSOSMC-DPC technique reduced ripples significantly compared to the DPC technique. In Ref.^35^, the author used both the DPC technique and indirect space vector modulation (ISVM) technique to improve the quality of the energy from the AG-WE system. The DPC-ISVM strategy is characterized by its ease and robustness compared to the DPC technique, as the simulation results proved the high efficiency of the DPC-ISVM technique in reducing current and torque fluctuations. All of these strategies mentioned significantly improve the characteristics of the DPC strategy, and this changes the structural structure of the traditional strategy, and this increases the complexity and difficulty of achieving experimental control, which is undesirable.

Another robust control scheme was used in^36^ to improve the current quality of a controlled AG-based wind turbine by DPC strategy, where the switching table and hysteresis comparators were compensated by the backstepping controller. The DPC technique based on backstepping control theory is more robust and is not affected by changing the parameters of the generator compared to the DPC technique. However, this method is characterized by its complexity and difficulty in achieving it compared to the DPC technique. However, the use of a backstepping controller in the DPC technique has greatly reduced the current and reactive power fluctuations compared to the DPC technique. There is another nonlinear technique that is simple, robust, easy to implement, and inexpensive compared to several nonlinear techniques such as SMC and backstepping control. This strategy is called synergetic control (SC), and this technique has been used in several fields^37–42^. This method reduces the phenomenon of chattering satisfactorily compared to the SMC and SC techniques. In Ref.^43^, the author used SC theory to improve the DPC strategy features of an AG-based dual-rotor WE system, where two SC controllers were used to control the active and reactive power. Among the advantages obtained by using the SC technique, are the improvement of the current quality, the reduction of the ripples of the effective and reactive power, and the improvement of the dynamic response of the reactive and active power compared to the traditional control. In Refs.^44,45^, two variants of the fractional-order super twisting algorithm were used to overcome the drawbacks of the DPC strategy and thus reduce the energy ripples of AG. The robustness of the generation system, which relies on a regular turbine, has been increased with the use of power estimation, which is undesirable, as it provides unsatisfactory results in the event of a malfunction in the machine. Also, a fuzzy super-twisting algorithm (FSTA) was used to control an AG inverter^46^. In this work, two FSTA controllers were used to control the power, and a modified SVM technique was used to generate the control pulses, and Matlab software was used to implement this control. The simulation results showed the high performance of the FSTA controller in the case of changing or not changing the parameters of the machine compared to the conventional control. In general, several new DPC technique strategies have been proposed to control AG using conventional and multi-rotor turbines to generate high-quality power while increasing durability. These new strategies are based on the use of modified SMC^47,48^, fractional-order control^49^, simplified super twisting sliding mode approaches^50^, third-order sliding mode^51^, and PD(1 + PI) technique^52^. All of these strategies are based on the classic structure of DPC, where both active and reactive power are estimated. The advantage of these strategies is robustness, simplicity, ease of implementation, and fast dynamic response. Moreover, in these specific strategies in the works^47–52^, the switching table is not used to generate control pulses, but the PWM technique is used for this purpose, which makes them uncomplicated strategies and maintains the advantage of simplicity that characterizes the traditional strategy. In Ref.^53^, a strategy for the DPC technique based on the use of both feedback PI controller and PWM technique is proposed to control the AG using a multi-rotor turbine, where simplicity and robustness are the main features of this strategy. This strategy was applied to a generator with a capacity of 1.5 MW, where three different tests were used to verify the behavior of the proposed DPC strategy. This strategy provided a fast dynamic response with fewer ripples for both active and reactive power compared to the DPC control. In addition, overshoot, steady-state error, time responses, feedback PI controllers give large reduction ratios for these values compared to conventional controls and some published scientific works. In Ref.^54^, neural PI controllers and a four-level neural modified SVM technique were used to overcome the drawbacks of the DPC technique, where a multi-level inverter was used to feed the instrument. In addition, a multi-rotor turbine is used to extract energy from wind energy and to greatly increase the stability of the system. Using Matlab software and operating the machine in different working conditions, the behavior of the proposed DPC strategy was verified, as it provided fast dynamic response and fewer ripples for both active and reactive power compared to DPC control. In addition, it provided excellent values for each of the overshoot, steady-state error, and time responses with high reduction ratios compared to the traditional control.

Some researchers suggested the use of smart techniques such as neural algorithms and fuzzy logic to minimize the ripples of the active power and current and improve the efficiency of the DPC technique. Several smart strategies have been used in the field of renewable energies, especially in both wind and solar energy^55–59^. Smart strategies such as fuzzy logic and neural networks do not need the mathematical form of the system under study, which makes them easy to use, and in the case of parameter changes, they provide excellent results. Also, it does not require a specialist and relies heavily on the experience of the user. In Ref.^60^, it is proposed to use neural networks in the place of the switching table of the DPC technique to obtain a signal at the output of the inverter with a fixed frequency. The use of neural networks improves the dynamic response and reduces active power fluctuations. Also, it is noticed that the current has a high quality in the case of using neural networks, and this is shown by the value of the current THD. In Ref.^61^, a new scheme for DPC using a neural PI (NPI) controller is proposed, where modified space vector modulation is used to control the generator inverter. This proposed DPC-NPI strategy is characterized by simplicity, low cost, fast dynamic response, and ease of implementation compared to the indirect FOC technique. The simulation results showed the characteristic of the DPC technique based on the NPI controller in reducing the active power and torque fluctuations and in improving the quality of the current. In Ref.^62^, two intelligent methods, fuzzy logic and the genetic algorithm (GA) were combined to improve the characteristics and advantages of the DPC technique for an AG-WE system. The combination of fuzzy logic and GA technique resulted in a more robust smart technique, and this is shown by simulation results in the case of changing the generated parameters, where we notice that the proposed intelligent DPC technique is not affected by the change of the generator parameters. In addition, the proposed intelligent DPC technique reduces the ripples of both active power and torque if the generator parameters are changed or not. A smart strategy represented by the GA technique was used in the work^63^ to calculate the terminal PI controller of the DPC strategy for the DFIG-based multi-rotor wind turbine (MRWT) system. The use of the GA technique leads to obtaining good results in terms of power ripples and current quality, and this is confirmed by the simulation results performed on a 1.5 MW AG compared to the traditional strategy of DPC. In Ref.^64^, the author calculated the control parameters of the DPC-PI technique using the GA technique, where using GA led to reduced power ripples and significantly improved current quality, which was confirmed by simulation results carried out on 1.5 MW A-based multi-rotor turbine systems. This negative strategy is represented by the large time it takes to calculate the system parameters, which is not desirable. Another intelligent strategy used in Ref.^64^ is particle swarm optimization (PSO). The latter was used to determine the parameters of the DPC-STA of AG-based wind turbines. By using digital simulations and under different operating conditions, the simulated results show that the DPC-STA strategy based on the PSO algorithm has a high performance in improving the system characteristics compared to the traditional control.

Through the results of these mentioned works, it is noted that the active power fluctuations remain and cannot be eliminated. Also, the current quality remains somewhat low depending on the type of controller (nonlinear or intelligent controller) used to improve the current quality. In this work, a new idea for the DPC technique is proposed by combining a PI controller, synergetic control, and genetic algorithm to obtain a more robust DPC technique while maintaining the simplicity of the classical DPC technique. The main contribution of the paper lies in integrating three different methods (PI controller, GA technique, and synergetic control) to obtain a robust and highly efficient controller for reducing the ripples of torque, current and active power of an asynchronous generator-based multi-rotor wind turbine system. The obtained strategy (synergetic-PI controller based on GA technique (SPI-GA)) is used to increase the efficiency of the DPC technique using the PWM strategy. This new strategy is different from many works^27,53,62,64^ in terms of principle, simplicity, ease, durability, and the results obtained. In addition, in this work, a multi-rotor turbine is used to generate the mechanical energy required to rotate the generator from the wind, which is a new technology that is not used in the above-mentioned works, except in some works that count on the fingers of the hand. Accordingly, which makes the paper of great importance in the field of scientific research. In addition, the use of the proposed synergetic-PI-GA controller in this work leads to the achievement of several other goals such as (1) Significantly reducing the ripples of the torque and active power, (2) Increasing the current quality to an excellent degree, (3) Decrease the THD value of the current, (4) Decrease the steady-state performance of the reactive and active power, (5) Obtaining a new smart nonlinear method that is not affected much by the change of generator parameters, as it can be proposed in the future to control electrical machines.

The rest of the work is organized as follows. Section “MRWT modeling” gives a model mathematical of the AG-based MRWT system. Section “Design of the SPI-GA controller” includes a detailed overview of the designed SPI-GA controller, where the pros and cons of the proposed DPC-SPI-GA strategy are mentioned. The designed DPC technique based on the SPI-GA controller is shown and compared with the DPC in Section “Proposed DPC-SPI-GA strategy”. In Section “Numerical results”, the numerical results using the Matlab software are presented and analyzed. The conclusion is the last part of this work done in this work, where the results obtained are extracted in the form of main points.

## MRWT modeling

**Figure 1:**
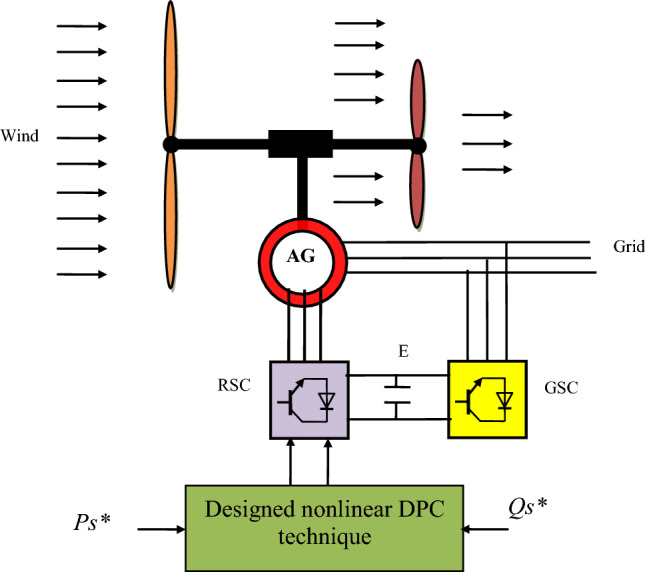
Structure of the MRWT system.

Figure 1 represents the wind system used in this work to generate electric power. It is noted that an MRWT is used to convert wind energy into mechanical energy. The use of MRWT increases the robustness and stability of the generation system while increasing the energy gained from the wind^64^. In addition, an AG is used to convert mechanical energy into electrical energy. In addition to using the AG, an AC-DC-AC inverter is used to feed the generator rotor from the network. In this type of generation system, the generator cannot be fed directly to the network, and an AC-DC-AC inverter is used to regulate the speed of the generator and the quality of the energy produced or the electric current. Therefore, it is necessary to choose a suitable technique for controlling the inverter.

To study any electrical power generation system, the mathematical form of both the turbine and the generator must be given using mathematical equations.

As mentioned earlier, MRWT was used to convert wind energy, as this turbine consists of two turbines of different sizes and power. The resulting power is the sum of the two capacities produced by the first and second turbines. In addition, the first turbine has more mechanical power generated than the second turbine and the same goes for torque. This technology is characterized by high efficiency, as it is expensive compared to conventional turbines^64^. The negative of this technology is that it contains a large number of mechanical components, which makes it difficult to control and difficult to maintain, which increases the industrial cost, and this is undesirable. Equation (1) represents both the torque and the mechanical power generated by the turbine used in this work^37^. In addition, the mechanical energy generated by the MRWT system is greater than the mechanical energy generated by the single-rotor wind turbine by about 23%, which is a very significant ratio and indicates the efficiency of the MRWT system^9^.

1 $$\left\{\begin{array}{l}{P}_{t}={P}_{1}+{P}_{2}\\ {T}_{t}={T}_{1}+{T}_{2}\end{array}\right.$$

where, *T*_*t*_ and *P*_*t*_ are the total torque and total mechanical power. *P*_*1*_ and *P*_*2*_ are the mechanical power of the first and second turbines., *T*_*1*_ and *T*_*2*_ are the torque of the first and second turbines.

The mechanical energies generated by the first and second turbines can be expressed by Eq. (2)^9,37^. The mechanical energy generated by the MRWT system is related to wind speed (*w*_*1*_ and *w*_*2*_), the coefficient of power (C_p_), air density (*ρ*), area(*S*_*1*_ and *S*_*2*_).

2 $$\left\{\begin{array}{l}{P}_{1}=\frac{{C}_{p}(\beta , \lambda )}{2}{S}_{1}\cdot \rho \cdot {w}_{1}^{3}\\ {P}_{2}=\frac{{C}_{p}(\beta , \lambda )}{2}{S}_{2}\cdot \rho \cdot {w}_{2}^{3}\end{array}\right.$$

Regarding the torque generated by each turbine, it is represented in Eq. (3), where the torque value is related to the wind speed, C_p_, ratio of speed (λ), and air density^11,12^.

3 $$\left\{\begin{array}{l}{T}_{1}=\frac{{C}_{p}(\beta , \lambda )}{2{\lambda }_{1}^{3}}{R}_{1}^{5}\cdot \rho \cdot \pi \cdot {w}_{1}^{2}\\ {T}_{2}=\frac{{C}_{p}(\beta , \lambda )}{2{\lambda }_{2}^{3}}{R}_{2}^{5}\cdot \rho \cdot \pi \cdot {w}_{2}^{2}\end{array}\right.$$

In the WE system, C_p_ is of great importance in calculating mechanical power and torque, as its value is related to tip speed ratio and pitch angle (*β*). The largest value for this parameter is in the case of *β* = 0°. This parameter is calculated according to Eq. (4)^37^.

4 $${C}_{p}\left(\beta , \lambda \right)=\frac{0.035}{{\beta }^{3}+1}+\frac{1}{0.08\beta +\lambda }$$

Each turbine has its own tip speed ratio, where the first turbine is used to control the MRWT system. Equation (5) represents the tip speed ratio of the first and second turbines, this value is related to the wind speed (*V*_1_) and the wind speed between the two turbines (*V*_2_).

5 $$\left\{\begin{array}{l}{\lambda }_{1}=\frac{{w}_{1}\cdot {R}_{1}}{{V}_{1}}\\ {\lambda }_{2}=\frac{{w}_{2}\cdot {R}_{2}}{{V}_{2}}\end{array}\right.$$

In the MRWT system used in this paper, the distance between the first and second turbines is estimated at 15 m, and to calculate the wind speed between the two turbines, Eq. (6) is used. This wind speed is related to the wind speed before the first turbine and the distance between the two turbines (*x*), which is estimated at 15 m. Also, the wind speed between the two turbines is related to a factor (C_T_) of 0.9^37^.

6 $${V}_{2}={V}_{2}\left(1-\frac{1-\sqrt{\left(1-{C}_{T}\right)}}{2}\left(1+\frac{2x}{\sqrt{1+4{x}^{2}}}\right)\right)$$

where *V*_1_ is the wind speed before the first turbine and *V*_2_ is the wind speed at any point between the two turbines.

The AG is an electrical machine that contains a fixed section called the stator and a moving section called the rotor, where each part can be expressed by mathematical equations. Equation (7) represents the voltage and flux of both the stator and the rotor of the generator^4,5^. Equation (8) represents the mechanical part of the AG. This equation expresses the relationship between speed and torque^27^.

7 $$\left\{\begin{array}{l}\begin{array}{c}\begin{array}{c}\begin{array}{c}{V}_{dr}={R}_{r}{I}_{dr}-{w}_{r}{\Psi }_{qr}+\frac{d}{dt}{\Psi }_{dr}\\ {V}_{qr}={R}_{r}{I}_{qr}+{w}_{r}{\Psi }_{dr}+\frac{d}{dt}{\Psi }_{qr}\end{array}\\ {\Psi }_{dr}={L}_{r}{I}_{dr}+M{I}_{ds}\\ {\Psi }_{qr}=M{I}_{qs}+{L}_{r}{I}_{qr}\end{array}\\ {V}_{ds}={R}_{s}{I}_{ds}-{w}_{s}{\Psi }_{qs}+\frac{d}{dt}{\Psi }_{sd}\\ {V}_{qs}={R}_{s}{I}_{qs}+{w}_{s}{\Psi }_{ds}+\frac{d}{dt}{\Psi }_{qs}\end{array}\\ {\Psi }_{qs}=M{I}_{qr}+{L}_{s}{I}_{qs}\\ {\Psi }_{ds}={L}_{s}{I}_{ds}+M{I}_{dr}\end{array}\right.$$

8 $$\left\{\begin{array}{l}{T}_{r}=1.5 p\times \frac{M}{{L}_{s}}(-{\Psi }_{sd}\times {I}_{rq}+{\Psi }_{sq}\times {I}_{rd})\\ {T}_{e}=J\times \frac{d\Omega }{dt}+f\times \Omega +{\mathrm{T}}_{\mathrm{r}}\end{array}\right.$$

An AG has two different capacities related to current and voltage. Equation (9) represents both the active and reactive power of the AG-based wind turbine system, where the value 1.5 expresses that the generator is a three-phase generator^13,20^.

9 $$\left\{\begin{array}{l}{Q}_{s}=1.5\times (- {I}_{qs}\times {V}_{ds}+{V}_{qs}\times {I}_{ds})\\ {P}_{s}=1.5\times ({V}_{qs}\times {I}_{qs}+ {I}_{ds}{\times V}_{ds})\end{array}\right.$$

In the next part, a new control technique is proposed for controlling the asynchronous generator, whereby both PI controller, GA technique, and SC theory are used to obtain a more robust strategy, improve the quality of the power and thus reduce the periodic maintenance.

## Design of the SPI-GA controller

In this part, a synergetic-PI controller based on the GA technique is presented as a new idea that was first introduced in this work to improve the advantages of the DPC strategy and to improve the quality of the power. So, three different methods are combined which are PI controller, GA technique, and SC theory to obtain a controller characterized by durability, ease, and effectiveness in reducing the active and reactive power fluctuations of an AG compared to several techniques such as SMC, PI controller, and SC technique. This proposed controller is different from many controllers^53,63,68^ in principle, simplicity, ease of tuning, and response to the presence of a small number of parameters, which helps to use artificial intelligence strategies such as the GA technique to calculate these parameters in a short time.

As it is known, the PI controller is one of the most popular and widely used strategies in the field of control and electronics due to its robustness and ease of implementation^65^. Compared with the hysteresis comparator, the PI controller features fast dynamic speed. But if the system parameters are changed, the PI controller is affected by this change and this is noticed in the presence of ripples and slow dynamic speed. Equation (10) represents the mathematical form of a PI controller, where K_i_ and K_p_ represent integral and proportional gains. These two parameters are used to adjust the response of the PI controller.

10 $${w(t)=K}_{p}\cdot S\left(t\right)+{K}_{i}.\int S\left(t\right)\cdot dt$$

where S(t) is the surface (S = X* − X).

The PI controller can be expressed by the transformation function represented in Eq. (11).

11 $$H\left(s\right)=\frac{w(s)}{S(s)}=\frac{{K}_{i}\cdot s+{K}_{p}}{s}$$

Traditionally, the SC technique is one of the simplest and easiest nonlinear techniques to apply to electrical machines^66^. This method is highly efficient compared to several methods such as the SMC technique, as it reduces the phenomenon of chattering significantly^67^. In addition to the simplicity and ease of implementation, the SC technique is considered one of the robust techniques that give satisfactory results in terms of improving dynamic response and reducing electric current ripples of the electric machine^37^. This strategy can be expressed by Eq. (12).

12 $$T\cdot \dot{S}+S=0$$

where T presents convergence speed, where the *T* > 0. *S* is the error (S = X* − X).

The solution to Eq. (12) is in the form represented in Eq. (13), where for t = 0, S(t) = S_0_. If t = T, in this case S(t) = S_0_·e, which is an exponential function.

13 $$S\left(t\right)={S}_{0}\cdot {e}^{t/T}$$

The SC strategy is known to be a stable technique, and Lyapunov’s theory can be used to check stability. Equation (14) represents the Lyapunov function used in this part to check the stability of the SC technique. Strategy function is considered as:

14 $$V=\frac{1}{2}\psi {(S)}^{2}$$

To study stability, the derivation of the Lyapunov function must be calculated, since the derivation of this function can be expressed by Eq. (15).

15 $$\dot{V}=\dot{\psi }\left(S\right)\cdot \psi (S)$$

As is known, in order for the technique to be stable, the derivation must be less than 0. So in this case we can write:

16 $$\dot{V}=-\frac{1}{K}{\psi }^{2}(S)\le 0$$

In the following, the new nonlinear controller that was proposed based on the use of both PI controller, GA technique, and SC strategy will be presented, where the GA strategy is used to calculate the parameters of the proposed synergetic-PI-GA (SPI-GA) controller. Equation (17) represents the mathematical form of the proposed SPI-GA control. The idea is to make both the PI controller and SC technique in series to obtain high efficiency. Among the advantages of the proposed SPI-GA controller are simplicity, low cost, ease of implementation, and durability compared to other techniques such as SMC and SC techniques.

17 $$H\left(t\right)=\left({K}_{1}\cdot \dot{S}+S\right)({K}_{2}\cdot S\left(t\right)+{K}_{3}\cdot \int S\left(t\right)\cdot dt)$$

Artificial intelligence techniques are among the solutions that are of great importance nowadays in the field of electrical machine control such as artificial neural algorithms, GA techniques, and fuzzy logic techniques to improve the characteristic of control strategies. The GA technique can be considered one of the oldest and most widely used of these techniques, as it is considered a mathematical technique and has been published in scientific articles for decades, and can be used in both nonlinear and linear systems^68,69^. This technique is used for calculating parameter values such as PI controller, where nominal values of PI controller parameters are obtained and operating in good conditions without excessive energy consumption^70^. On the other hand, the GA technique was used to calculate the parameters of the proposed nonlinear controller to obtain normal operation without excessive energy consumption. Also, the proposed nonlinear controller has a better dynamic response compared to the traditional controller (hysteresis comparator or PI) due to the use of the GA technique.

**Figure 2:**
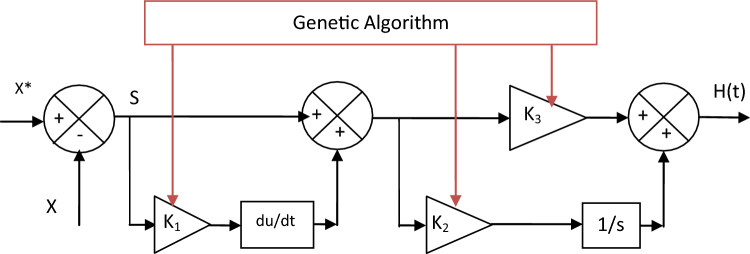
Designed SPI-GA controller.

The proposed SPI-GA controller can be expressed or represented by a diagram to facilitate understanding and simplify the meaning. SPI-GA controller does not need to know the mathematical form of the system under study, which makes it provide satisfactory results and performance in case of system parameters change, and this is desirable. The characteristics of the genetic algorithm used in this work to calculate the parameters of the proposed controller are found in the Appendix section, where all the necessary information and characteristics necessary to implement this smart strategy are recorded. Figure 2 represents the proposed SPI-GA controller in this work, as this control was proposed for the first time in this work as a better solution to improve the quality of the energy and reduce the torque and current fluctuations. As shown in Fig. 2, the SPI-GA controller is different from several controllers such as PD(1 + PI) controller. Also, this proposed controller is classified into linear controllers that have high performance and efficiency in improving the performance of systems, and this is confirmed by the fifth part of this work (Table S1).

To study the stability of the proposed SPI-GA controller, the Lyapunov theorem can be used and the derivation of the Lyapunov function is calculated, and the stability condition is that the derivative of the Lyapunov function is negative (less than 0).

**Figure 3:**
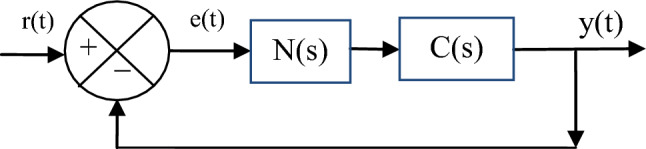
A block diagram of a SPI-GA regulator in a feedback loop.

To study the stability of the designed SPI-GA controller used to control a system defined by a function C(s). For this purpose, Fig. 3 is used. The function N(s) expresses the transformation function of the proposed SPI-GA controller.

where, y(t) is the measured process, r(t) is the desired process, and C(s) is process transfer function.

The total loop transfer function is shown in Eq. (18).

18 $$H(s)=\frac{N\left(s\right)\cdot C(s)}{1+N\left(s\right)\cdot C(s)}$$

The system represented in Fig. 3 is unstable if the transformation function H(s) diverges for some s. The system is stable if the conversion function H(s) converges to s. Thus the system under study is unstable in cases where N(s)·C(s) = − 1 with $\left|\mathrm{N}\left(\mathrm{s}\right)\cdot \mathrm{C}(\mathrm{s})\right|$ = 1. For the system under study to be stable, the condition N(s)·C(s) < 1 must be met. Moreover, the Nyquist stability theory can be used to study the stability of the designed SPI-GA controller.

## Proposed DPC-SPI-GA strategy

**Figure 4:**
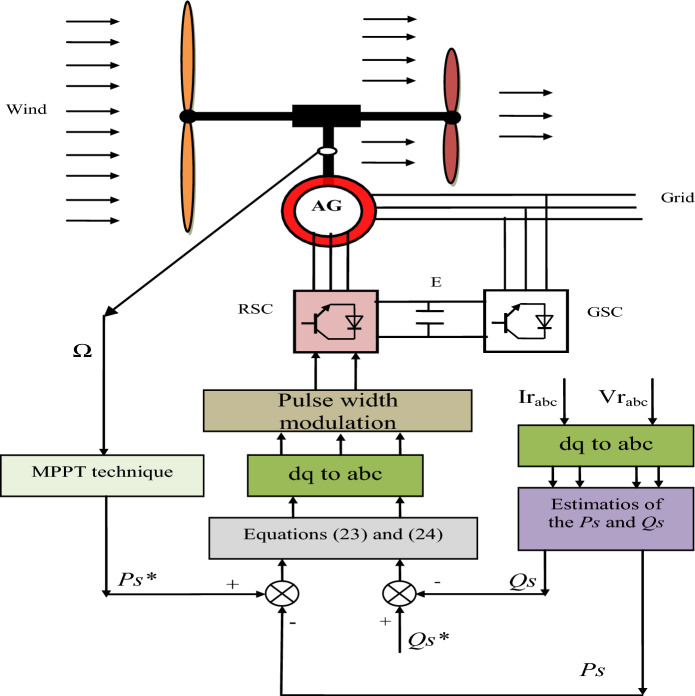
The designed DPC-SPI-GA strategy of the AG-MRWT system.

The reactive and active power ripples are two of the biggest problems and disadvantages of the DPC strategy due to the use of hysteresis comparators and switching tables^71^. However, the quality of the current is low, as the current at the output of the inverter contains many ripples, which causes disturbances in the operation of the devices and irregular motor’s speed. To avoid and overcome these shortcomings, a new control theory is put forward for electric power generation using the AG-MRWT system. The idea is to use the technique proposed in Section “Design of the SPI-GA controller” to control the reactive and active power of the AG-MRWT system. Figure 4 represents the proposed control scheme for controlling the AG-MRWT system. By this figure, the proposed control scheme is a modification of the classical DPC technique, where two SPI-GA controllers are used in place of the hysteresis comparator. In this proposed control scheme, the PWM technique is used to control the AG inverter due to its simplicity, low cost, inexpensive maintenance, and ease of implementation. Also, the SVM technique can be used in this proposed control scheme in place of using the PWM technique to control the AG inverter. In this proposed control scheme, the simplicity and ease of implementation that characterize the classical DPC technique were preserved, and in return, the degree of durability was increased, due to the use of the SPI-GA controller. Also, the proposed control aims to calculate the reference voltage values based on the error in the active and reactive power. As in the case of the traditional strategy, the estimation of the active and reactive power is used in the proposed control to calculate the error in the capacity. Therefore, the same estimation equations used in conventional control are used.

To control the powers, both the reactive power and the active power are estimated to calculate the error in the powers. Therefore, measurement of voltage and current is necessary to calculate the rotor and stator fluxes. As is known, to estimate the *Qs* and *Ps*, current, flux, rotor and stator resistances and voltage must be known. The flux is calculated according to Eq. (19)^72^.

19 $$\left\{\begin{array}{l}\left|{\Psi }_{r}\right|=\sqrt{\left({\Psi }_{r\beta }^{2}+{\Psi }_{r\alpha }^{2}\right)}\\ \left|{\Psi }_{s}\right|=\sqrt{\left({\Psi }_{s\beta }^{2}+{\Psi }_{s\alpha }^{2}\right)}\end{array}\right.$$

With:

20 $$\left\{\genfrac{}{}{0pt}{}{{\Psi }_{r\beta }={\int }_{0}^{t}({V}_{r}-{R}_{r}\times {i}_{r\beta })dt}{{\Psi }_{r\beta }={\int }_{0}^{t}({V}_{r}-{R}_{r}\times {i}_{r\beta })dt}\right.$$

21 $$\left\{\genfrac{}{}{0pt}{}{{\Psi }_{s\beta }={\int }_{0}^{t}({V}_{s}-{R}_{s}\times {i}_{s\beta })dt}{{\Psi }_{s\beta }={\int }_{0}^{t}({V}_{s}-{R}_{s}\times {i}_{s\beta })dt}\right.$$

Using Eqs. (19), (20), and (21) it is possible to estimate both the *Qs* and *Ps* according to the following equation^73^:

22 $$\left\{\begin{array}{l}{Q}_{s}=-\frac{3}{2}\left(\frac{{V}_{s}}{{\sigma \times L}_{s}}{\times \Psi }_{\beta r}-\frac{{V}_{s}{\times L}_{m}}{{\sigma {\times L}_{r}\times L}_{s}}\right)\\ {P}_{s}=-\frac{3}{2}{V}_{s}{\times \Psi }_{r\beta }\times \frac{{L}_{m}}{{\sigma \times {L}_{r}\times L}_{s}}\end{array}\right.$$

The proposed DPC-SPI-GA technique aims to calculate the reference values of both direct and quadrature rotor voltage using both active and reactive power errors. Equations (23) and (24) represent both direct and quadrature voltage references used to extract three-phase voltage.

23 $${V}_{dr}^{*}=\left({K}_{1}\cdot \dot{{S}_{Ps}}+{S}_{Ps}\right)({K}_{2}\cdot {S}_{Ps}\left(t\right)+{K}_{3}\cdot \int {S}_{Ps}\left(t\right)\cdot dt)$$

24 $${V}_{qr}^{*}=\left({K}_{1}\cdot \dot{{S}_{Qs}}+{S}_{Qs}\right)({K}_{2}\cdot {S}_{Qs}\left(t\right)+{K}_{3}\cdot \int {S}_{Qs}\left(t\right)\cdot dt)$$

**Figure 5:**
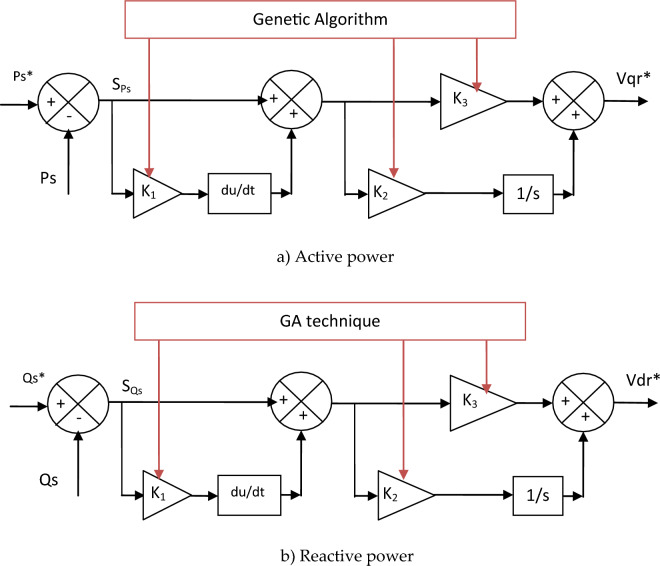
Proposed reactive and active power controllers.

Equations (23) and (24) can be simulated by Fig. 5 to simplify understanding and give an accurate picture of exactly the work done in this paper. Through this figure, it is noted that the proposed control is simpler and not related to the system (parameters), which makes it give better results in case of changing the system parameters.

Regarding the reference value of the active power (Ps*), this value is calculated using the maximum power point tracking (MPPT) technique and it is related to the wind speed. The reference value of the reactive power (Qs*) is set to 0 VAR.

The proposed DPC-SPI-GA technique has the characteristics and advantages of the classical DPC strategy, as they are similar in the degree of simplicity and ease of implementation. However, it differs from the classical DPC strategy in terms of durability and ripple values for both torque and current. In Table 1, the similarities and differences between the two techniques are summarized.

**Table 1:** A comparative study between the DPC-SPI-GA and DPC strategies.

	DPC	DPC-SPI-GA
Switching table	Yes	No
Current quality	Medium	High
Simplicity	Yes	Yes
Measure current and voltage	Yes	Yes
Robustness	Low	High
Active power ripple	High	Low
Park transformation	Yes	Yes
Active power response dynamic	Slow	Quick
Pulse width modulation	No	Yes
Power estimation	Yes	Yes
Hysteresis comparator	Yes	No

## Numerical results

In this part, the DPC-SPI-GA technique is achieved using Matlab software, where the DPC-SPI-GA technique is compared with the DPC technique from several aspects such as the value and ratios of current and active power ripples, steady-state error (SSE), and THD of the current. Moreover, three tests are proposed to verify the effectiveness of the DPC-SPI-GA technique compared to the DPC strategy. Also, an AG-MRWT with a capacity of 1.5 MW is used, which is placed in the MRWT system. The AG parameters used in this paper are as follows:* R*_*s*_ = 0.012 Ω, *P*_*sn*_ = 1.5 MW, *L*_*r*_ = 0.0136 H, *L*_*m*_ = 0.0135 H, *J* = 1000 kg m^2^, 380/696 V, *R*_*r*_ = 0.021 Ω, 50 Hz, *L*_*s*_ = 0.0137 H, p = 2, and *f*_*r*_ = 0.0024 N m/s ^43,64^.

### First test

**Figure 6:**
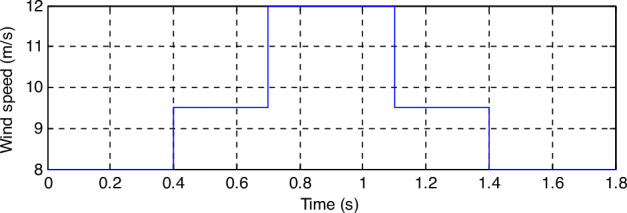
Steps speed wind profile.

**Figure 7:**
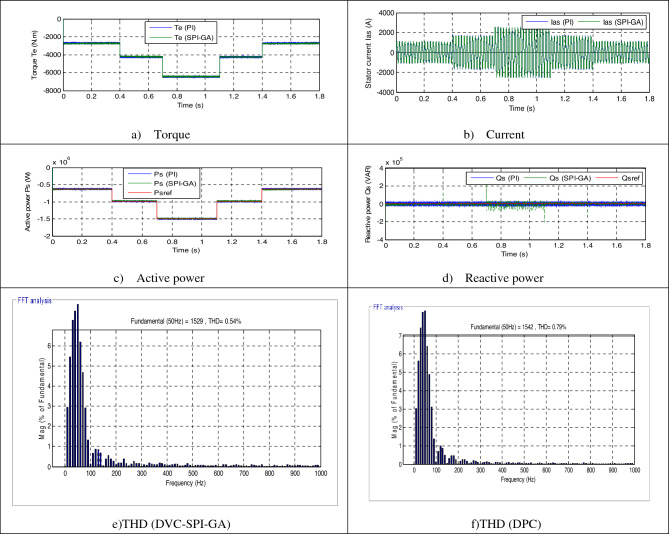
First test results.

In this part, the characteristic of the designed DPC-SPI-GA technique is tested compared to the DPC technique in the case of using steps wind speed, where the results obtained are represented in Fig. 7. Also, the wind speed used in this test is represented in Fig. 6. Through Fig. 7c, the active power takes the form of the wind speed, which indicates that the active power is affected by the shape of the wind speed. In addition, the reactive power is not affected by the change in wind speed and remains nil regardless of the change in wind speed (see Fig. 7d). From Fig. 7a and b, it can be said that current and torque have the form of a change in wind speed, as their value is affected by a change in wind speed.

**Figure 8:**
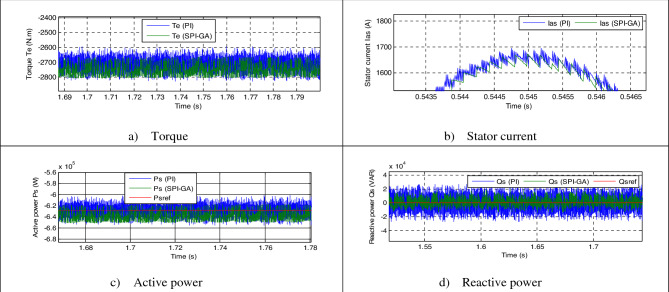
Zoom in the first test.

Figure 8 represents the zoom in the torque, reactive power, current and active power of both strategies. The value of the ripples of the current, active power, torque, and reactive power are shown in Table 2, where it is noted that the DPC-SPI-GA reduced the value of the ripples compared to the DPC strategy and the reduction ratio was about 34%, 22.95%, 44%, and 36.93% for each of the torque, *Ps*, current and *Qs*, respectively.

**Table 2:** The ripple value of the DPC and DPC-SPI-GA strategies (First test).

	*Qs* (VAR)	*Te* (N m)	*Ps* (W)	*Ias* (A)
DPC	48,000	200	45,000	75
DPC-SPI-GA	30,272	132	34,672	42
Ratios	36.93%	34%	22.95%	44%

Figure 7f and e represent the THD value of the current for each of the DPC-SPI-GA technique (0.54%) and DPC technique (0.79%), respectively, as the DPC-SPI-GA technique minimized the THD by about 31.64% compared to the DPC technique. So, it can be said that the DPC-SPI-GA technique is better than the DPC technique, and this thing will be confirmed in the next test. The negative of the proposed control lies in the amplitude of the fundamental (50 Hz) signal of current, where the value of the amplitude was 1542 A for the conventional control and 1529 for the proposed control. The main advantage of this proposed control scheme is that the percentage of SSE for *Ps* and *Qs* are reduced about 35% and 36.93%, respectively, compared to the DPC technique (see Table 3).

**Table 3:** The SSE value of the reactive/active power (First test).

	*Qs* (VAR)	*Ps* (W)
DPC	24,000	20,000
DPC-SPI-GA	15,136	13,000
Ratios	36.93%	35%

### Second test

**Figure 9:**
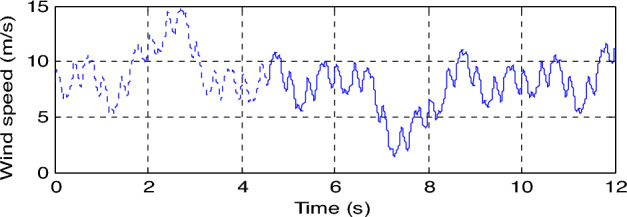
Wind speed profile.

**Figure 10:**
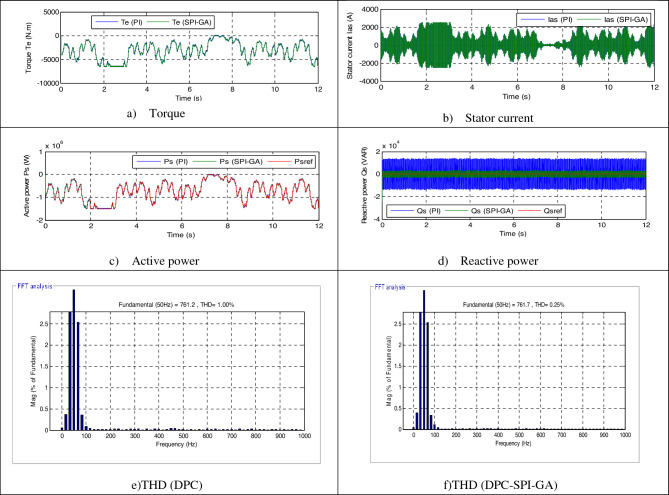
Second test results.

In this section, the behavior of the DPC-SPI-GA technique will be studied compared to the DPC technique in the case of variable wind speed, where a variable wind speed is used and takes the form shown in Fig. 9. The obtained results are shown in Fig. 10. Through this figure, the *Qs* and *Ps* follow the references (Qs* and Ps*) in an excellent manner (see Fig. 10c and d) with a preference for the suggested strategy in terms of SSE value and dynamic response of the *Qs* and *Ps*. Also, the active power changes according to the change in wind speed, while the reactive power remains constant (zero) and is not affected by the change in wind speed. On the other hand, current and torque change according to the change in wind speed, as the greater the wind speed, the greater the value of both current and torque (see Fig. 8a and b).

Figure 8e and f represent the THD of the current for each of the DPC and DPC-SPI-GA techniques, respectively, where the THD value in the case of the DPC-SPI-GA technique was 0.29% and 1.00% for the DPC technique. Therefore, it can be said that the DPC-SPI-GA technique is better in terms of the THD value of about 71%. On the other hand, the proposed control presented a greater amplitude of the signal of fundamental (50 Hz) current compared to the conventional control, where the value of the amplitude was 761.2 A and 761.7 A for both the conventional control and the proposed control, respectively.

**Figure 11:**
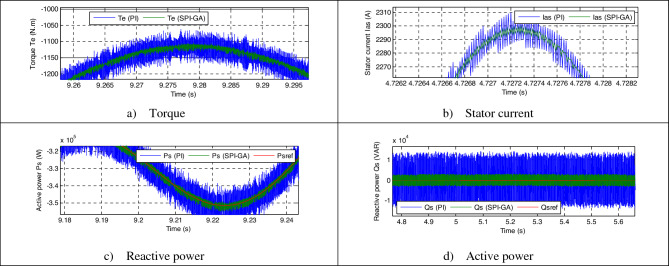
Zoom in the second test.

Figure 11 represents the zoom in the torque, reactive power, current and active power of both strategies. The DPC-SPI-GA technique presented greater ripples compared to the DPC technique, where the values of the torque, *Qs*, current, and *Ps* are shown in Table 4. Through this table, the DPC-SPI-GA technique is better than the DPC technique in terms of reducing ripples and the reduction ratios were 79.16%, 78.12%, 73.66%, and 86% for torque, *Qs*, current, and *Ps*, respectively.

**Table 4:** The ripple value of the DPC and DPC-SPI-GA strategies (Second test).

	*Qs* (VAR)	*Te* (N m)	*Ps* (W)	*Ias* (A)
DPC	28,000	120	25,000	30
DPC-SPI-GA	6124	25	3500	7
Ratios	78.12%	79.16%	86%	73.66%

To prove the effect of the proposed DPC-SPI-GA strategy on the generation system, the SSE value was extracted for both the *Ps* and *Qs*, where results were recorded in Table 5. The SSE value for the active power was 10,000 W for the classical technique and 1320 W for the proposed DPC-SPI-GA technique, which makes the proposed DPC-SPI-GA technique the best in terms of SSE value, and the estimated reduction ratio is about 86.80%. Also, Table 4 shows that the DPC-SPI-GA technique provided a better value for SSE compared to the DPC technique, and a reduction was estimated at 78.12%. So, it can be said that the DPC-SPI-GA technique is the best and most efficient solution because the reduction ratios for both ripples and SSE value were very-high.

**Table 5:** The SSE value of the reactive/active power (Second test).

	*Qs* (VAR)	*Ps* (W)
DPC	14,000	10,000
DPC-SPI-GA	3062	1320
Ratios	78.12%	86.8%

### Third test

This test aims to study the durability of the proposed control compared to the traditional control, where the same form of wind speed change used in the second test is used. A comparison study is made between the two controls in terms of ripple ratio, current quality, trace references, THD of current, SSE value, …etc.

**Figure 12:**
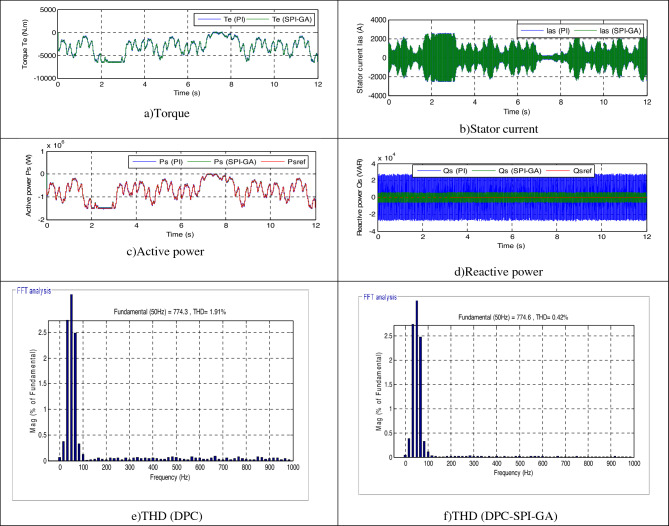
Third test results.

**Figure 13:**
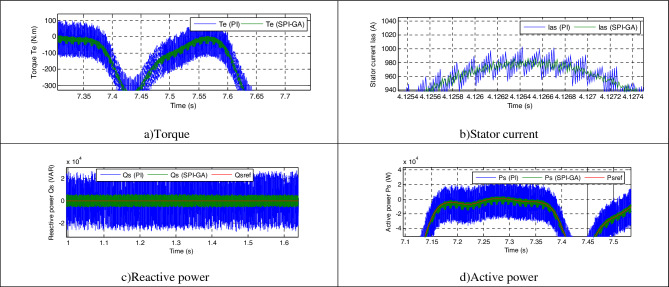
Zoom in the third test.

In this test, the generated parameters (*Rs*, *Lr*, *Lm*, *Rr*, and *Ls*) are changed to new values (see Table 6), where the behavior of the DPC-SPI-GA technique is studied compared to the DPC technique. The obtained results are shown in Fig. 12. Through this figure, the *Qs* and *Ps* follow the references (Qs* and Ps*) well, with always preference for the DPC-SPI-GA technique in terms of SSE value and response dynamic. Torque and current remain in the form of active power, whereby the higher the active power, the greater the value of both current and torque (see Fig. 12a,b). Also, more fluctuations are observed at the level of current, active power, torque, and reactive power in the case of the DPC technique compared to the DPC-SPI-GA technique (see Table 7). Figure 13 represents the zoom in the torque, *Qs*, current, and *Ps* of both strategies. Accordingly, it can be said that the DPC-SPI-GA technique minimized the ripples of torque, *Qs*, current, and *Ps* by 67.50%, 78.42%, 77.61%, and 80.18% respectively, compared to the DPC technique.

**Table 6:** New values for the AG parameters.

*Rs*	*Ls*	*Lm*	*Rr*	*Lr*
0.024 Ω	0.00685 H	0.00675 H	0.042 Ω	0.0068 H

**Table 7:** The ripple value of the both strategies (Third test).

	*Qs* (VAR)	*Te* (N m)	*Ps* (W)	*Ias* (A)
DPC	56,854	160	53,000	67
DPC-SPI-GA	12,268	52	10,500	15
Ratios	78.42%	67.50%	80.18%	77.61%

The current THD was 1.91% for the traditional DPC strategy and 0.42% for the DPC-SPI-GA technique (see Fig. 12f,e). Through these two values, the DPC-SPI-GA technique reduced the THD value by 78.01% compared to the DPC technique. In addition, the DPC-SPI-GA technique presented a greater amplitude of the signal of fundamental (50 Hz) current compared to the DPC technique, where the value of the amplitude was 774.3 A and 774.6 A for both the DPC and the DPC-SPI-GA techniques, respectively. Through these obtained results, it can be said that the DPC-SPI-GA technique is the best solution for controlling the AG-MRWT system because of its robustness, and simplicity and the results obtained. Moreover, the SSE value in the DPC-SPI-GA technique is much smaller than the DPC strategy, where the reduction ratios were estimated at 78.42% and 85% for each of the reactive and active power, respectively (see Table 8).

**Table 8:** The SSE value of the reactive/active power (Third test).

	*Qs* (VAR)	*Ps* (W)
DPC	28,427	20,000
DPC-SPI-GA	6134	3000
Ratios	78.42%	85%

Through the three tests, the DPC-SPI-GA technique is better than the DPC technique. In the following, the DPC-SPI-GA technique will be compared with some published papers in terms of the THD of the current. The results of the comparison are recorded in Table 9. To this table, the DPC-SPI-GA technique is the best solution for reducing the THD and thus improving the quality of the current compared to several strategies such as DPC and DTC techniques. Therefore, it can be said that this strategy is the best solution for controlling electric generators.

**Table 9:** A comparative study between the DPC-SPI-GA and other published papers in terms of the THD.

References	Strategies		THD (%)
^74^	DPC-IP strategy		0.43
^75^	Nonlinear DVC strategy		0.50
^76^	FOC technique		3.7
^77^	Indirect FOC technique		6.5
^78^	DPC with STA controller		1.66
^79^	Power control	Technique 1	5.6817
		Technique 2	3.1873
^80^	12 Sectors DPC strategy		0.40
^81^	DTC		6.70
	Fuzzy DTC		2.40
^82^	DTC technique		7.83
	Neural DTC strategy		3.26
^83^	SOSM technique		3.13
^84^	DTC using L-filter		10.79
	DPC using LCL-filter		4.05
^85^	Integral SMC technique		9.71
	Multi-resonant-based SMC technique		3.14
^70^	DPC based on Passivity controller		2.51
^86^	2-Level DTC		8.75
	3-Level DTC		1.57
^87^	GA-least squares wavelet support vector machines strategy		3.39
Designed DPC-SPI-GA strategy	First test		0.54
	Second test		0.29
	Third test		0.42

The results of the second test are taken where the wind speed is variable and compared with other published scientific works in terms of the ripple reduction ratio for each of the capabilities, torque, and current. The results are recorded in Table 10. Through this table, the proposed control in this work presented excellent reduction ratios greater than the reduction ratios for several strategies, which indicates its high efficiency and ability to significantly improve system properties, which is desirable in the field of control.

**Table 10:** Comparison in terms of ripple reduction rates.

References		Ratios			
		*Ps* (W), %	*Qs* (VAR), %	*Ias* (A), %	*Te* (N m), %
^88^		41.17	94	50	40
^89^	STA	21.75	22.66	18.13	16.31
	MSTA	19.11	21.23	20.17	15.39
^90^	DPC-ANN	45.26	66.29	–	–
	DPC-NF	57.74	67.13	–	–
^91^	Backstepping control	28.57	46.93	65	–
^92^	Intelligent control	36	35	63.75	–
^52^	DPC-PD(1 + PI)	47.50	46.68	–	–
^63^	DPC-SPI-GA	81	75.98	70.21	60
^53^	Feedback PI control	96.65	37.14	59.25	63.33
Proposed technique		86	78.12	73.66	79.16

In Table 11, a comparative study is carried out between the proposed control in this work and some other works in terms of the SSE value of the active and reactive power. This table gives a clear picture of the superiority of the proposed control over some strategies, as this superiority appears through the high reduction ratios, and this is a desirable thing that indicates high efficiency. Moreover, the results of this table confirm the results of Tables 9 and 10, which increases the reliability of the proposed control.

**Table 11:** Comparison in terms of SSE reduction rates.

References		Ratios	
		*Qs* (VAR), %	*Ps* (W), %
^53^	Feedback PI controller	45.48	78
^91^	Backstepping control	82.70	50
^92^	Intelligent control	35.48	62
^52^	DPC-PD(1 + PI)	78.44	45.83
^63^	DPC-SPI-GA	75.98	83.33
Proposed technique		78.12	86.8

## Conclusion

This work presents a new technique based on the use of a synergetic-PI-GA controller, applied to the AG-MRWT system, which greatly increases the robustness of the system and improves the current quality of the electrical grid. The proposed technique based on the SPI-GA controller is a simple and robust strategy. Also, Ease of implementation is one of the biggest advantages of the proposed method. This technique is suggested to reduce the reactive and active power fluctuations and increase the quality of electrical power in the distribution networks. SPI-GA controller was introduced as a solution for controlling asynchronous generators due to its ease, durability, and simplicity. So SPI-GA controller is an effective tool that can be proposed and used for controlling very complex and non-linear systems.

This proposed strategy based on the SPI-GA controller, it led to a better current source if the generator parameters were changed, as the reduction ratio for the THD value was about 71% compared to the DPC technique. Also, the designed control scheme reduced the ripples of *Ps*, current, torque, and *Qs* by estimated ratios of 86%, 73.66%, 79.16%, and 78.12%, respectively. Therefore, the results obtained from this work can be summarized in the following points:

- The numerical results confirmed the robustness, efficacity, and validity of the proposed synergetic-PI-GA controller compared to the traditional PI controller.
- The designed synergetic-PI-GA controller minimized the SSE of the reactive/active power compared to the PI controller.
- Reduce the THD value of the current.
- The use of the proposed synergetic-PI-GA controller increases the durability of the PI controller and helps improve its properties.
- The DPC-SPI-GA technique is better than the DPC technique of the AG-MRWT system.
- The DPC-SPI-GA strategy improves the quality of the current and reactive/active power of the AG-MRWT system.

In the next work, a backstepping control theory based on fractional calculus and PSO algorithm will be designed to control the multi-rotor wind turbine system, where the asynchronous generator was controlled using direct vector control.

### Supplementary Information

Supplementary Table S1.

## Data Availability

Data available on request from the authors. The datasets used and/or analysed during the current study available from the corresponding author on reasonable request. In the event of communication, the first author (Habib Benbouhenni, E-mail: habib.benbouenni@nisantasi.edu.tr) will respond to any inquiry or request.
