# Supplementary material for: Synergetic-PI controller based on genetic algorithm for DPC-PWM strategy of a multi-rotor wind power system
Source: Sci Rep. 2023 Aug 21;13:13570. doi: 10.1038/s41598-023-40870-7 (PMC10442353; doi:10.1038/s41598-023-40870-7)
Supplement: Supplementary file 1 — Supplementary Table S1. [file 41598_2023_40870_MOESM1_ESM.docx]

Supplementary Material: Appendix section

**Appendix**

Table S1 represents the characteristics of the GA technique used to calculate the parameters of the designed controller.

**Table S1.** Parameters of GA technique

| **Current iteration** | | | | 51 |
| --- | --- | --- | --- | --- |
| **Mutation : Mutation function** | | | | Use constraint dependent defaut |
| **Hybrid function : hybrid function** | | | | None |
| **User function evaluation** | | | | Evaluate fitness and constraint functions : in serial |
| **Population** | | **Creation function** | | Use constraint dependant defaut |
|  |  | **Population size** | | Use default : 20 |
|  |  | **Population type** | | Double vector |
| **Fitness scaling : Scaling function** | | | | Rank |
| **Selection : Selection function** | | | | Stochastic uniform |
| **Reproduction** | | | **Cross over fraction** | Use default : 0.8 |
|  |  |  | **Elite count** | Use default : 2 |
| **Bounds : lower** | | | | [-10 -100 0] [200 2000 0] |
| **Number of variable** | | | | 3 |
| **Output function : History to new window** | | | | Interval : 1 |
| **Migration** | **Fraction** | | | Use default : 0.2 |
|  | **Interval** | | | Use default : 20 |
|  | **Direction** | | | Forward |
| **Algorithm settings** | **Intial penalty** | | | Use default : 10 |
|  | **Penalty factor** | | | Use default : 100 |
| **Display to command window** | | | | Level of display : off |
| **Stopping criteria** | **Generations** | | | Use default : 100 |
|  | **Stall generations** | | | Use default : 50 |
|  | **Stall time limit** | | | Use default : inf |
|  | **Fitness limit** | | | Use default : -inf |
|  | **Stall generations** | | | Use default : 50 |
|  | **Function tolerance** | | | Use default : 1e-6 |
|  | **Stall generations** | | | Use default : 50 |
|  | **Nonlinear constraint tolerance** | | | Use default : 1e-6 |
| **Crossover : crossover function** | | | | Scattered |
